# A pilot randomized controlled trial comparing THUNDERBEAT to the Maryland LigaSure energy device in laparoscopic left colon surgery

**DOI:** 10.1007/s00464-021-08765-8

**Published:** 2021-11-01

**Authors:** Jeffrey W. Milsom, Koianka Trencheva, Kota Momose, Miroslav P. Peev, Paul Christos, Parul J. Shukla, Kelly Garrett

**Affiliations:** 1 Section of Colorectal Surgery, Department of Surgery, Weill Cornell Medicine College, New York, NY 10065 USA; 2 Division of Biostatistics and Epidemiology, Department of Healthcare Policy & Research, Weill Cornell Medicine, New York, NY USA

**Keywords:** THUNDERBEAT, Surgery, Energy devices

## Abstract

**Background:**

The THUNDERBEAT is a multi-functional energy device which delivers both ultrasonic and bipolar energy, but there are no randomized trials which can provide more rigorous evaluation of the clinical performance of THUNDERBEAT compared to other energy-based devices in colorectal surgery. The aim of this study was to compare the clinical performance of THUNDERBEAT energy device to Maryland LigaSure in patients undergoing left laparoscopic colectomy.

**Methods:**

Prospective randomized trial with two groups: Group 1 THUNDERBEAT and Group 2 LigaSure in a single university hospital. 60 Subjects, male and female, of age 18 years and above undergoing left colectomy for cancer or diverticulitis were included. The primary outcome was dissection time to specimen removal (DTSR) measured in minutes from the start of colon mobilization to specimen removal from the abdominal cavity. Versatility (composite of five variables) was measured by a score system from 1 to 5 (1 being worst and 5 the best), and adjusted/weighted by coefficient of importance with distribution of the importance as follow: hemostasis 0.275, sealing 0.275, cutting 0.2, dissection 0.15, and tissue manipulation 0.1. Other variables were: dryness of surgical field, intraoperative and postoperative complications, and mortality. Follow-up time was 30 days.

**Results:**

60 Patients completed surgery, 31 in Group 1 and 29 in Group 2. There was no difference in the DTSR between the groups, 91 min vs. 77 min (*p* = 0.214). THUNDERBEAT showed significantly higher score in dissecting and tissue manipulation in segment 3 (omental dissection), and in overall versatility score (*p* = 0.007) as well as versatility score in Segment 2 (retroperitoneal dissection *p* = 0.040) and Segment 3 (*p* = 0.040). No other differences were noted between the groups.

**Conclusions:**

Both energy devices can be employed effectively and safely in dividing soft tissue and sealing mesenteric blood vessels during laparoscopic left colon surgery, with THUNDERBEAT demonstrating some advantages over LigaSure during omental dissection and tissue manipulation.

ClinicalTrial.gov # NCT02628093.

The growing use of laparoscopic surgery for benign and malignant colorectal diseases during the last two decades has prompted development of new small caliber (5 mm) electrosurgical instruments for safe and effective hemostasis, vessel sealing, and tissue dissection [1–3]. Furthermore, the aim of these energy devices has also been to shorten operative time, lessen the thermal spread, and reduce the need for instrument exchange. The currently available energy devices used during laparoscopic colorectal surgery utilize three different energy-based methods: monopolar (ME), bipolar (BE) and ultrasonically activated electrosurgery (UAS) (4-Milsom). Amongst the most generally available energy devices in the USA are LigaSure™ and Sonicision, Medtronic, USA; Harmonic Ace, Ethicon Endo-Surgery, USA; and THUNDERBEAT (TB), Olympus, Japan. All are insertable into the abdominal cavity via 5 mm port. The TB is a multi-functional energy device which delivers simultaneously ultrasonic and bipolar energy. This allows surgeons to seal and safely divide blood vessels up to 7 mm, cut and dissect omental and mesentery tissue, and potentially reduce the need for instrument exchange [2, 4, 5]. UAS devices are multi-functional similarly to TB, but are approved to only seal vessels up to 4–5 mm in diameter [6, 7]. Bipolar electrosurgical technology has been widely used in laparoscopic bowel resection and considered a safe method for dissection and vessel ligation [1, 8–10]. Recent studies comparing TB to other energy devices suggest that all are safe and effective to use in laparoscopic colorectal surgery and report similar intraoperative and postoperative outcomes [2, 11, 12]. However, most of these studies are retrospective cohort studies or small prospective cohorts. There are no randomized controls trials which can provide more rigorous evaluation of the clinical performance of TB compared to other energy-based devices in laparoscopic colorectal surgery.

## Aims/objective

The aim of this randomized trial was to compare the clinical performance between the THUNDERBEAT and Maryland LigaSure Energy Devices in performing soft tissue dissection, dividing and sealing blood vessels in patients undergoing left laparoscopic colectomy.

## Materials and methods

### Study design

This was a pilot prospective randomized trial in a single academic institution. Patients undergoing laparoscopic left colectomy for their medical condition were randomized with equal chances into one of two groups: Group 1—THUNDERBEAT and Group 2—LigaSure. After randomization, the study involved prospective data collection before, during and after surgery and video recording of the surgery. All surgeries were carried out according to the regular surgical and anesthesia care. The follow up after surgery was 30 days. The study was approved by the Institutional Review Board.

### Population

Sixty patients, male and female, age 18 years and above and American Society of Anesthesiologists (ASA) class between 1 and 3, undergoing elective left laparoscopic colectomy for neoplasm or diverticulitis, were included in the study after providing research informed consent. Patients with morbid obesity (body mass index, BMI > 35), multiple previous abdominal surgeries, on anticoagulants prior to surgery, coagulopathy disorders, pregnant women, and those to whom electrosurgery is contraindicated were excluded from the study.

### Outcomes

**Fig. 1:**
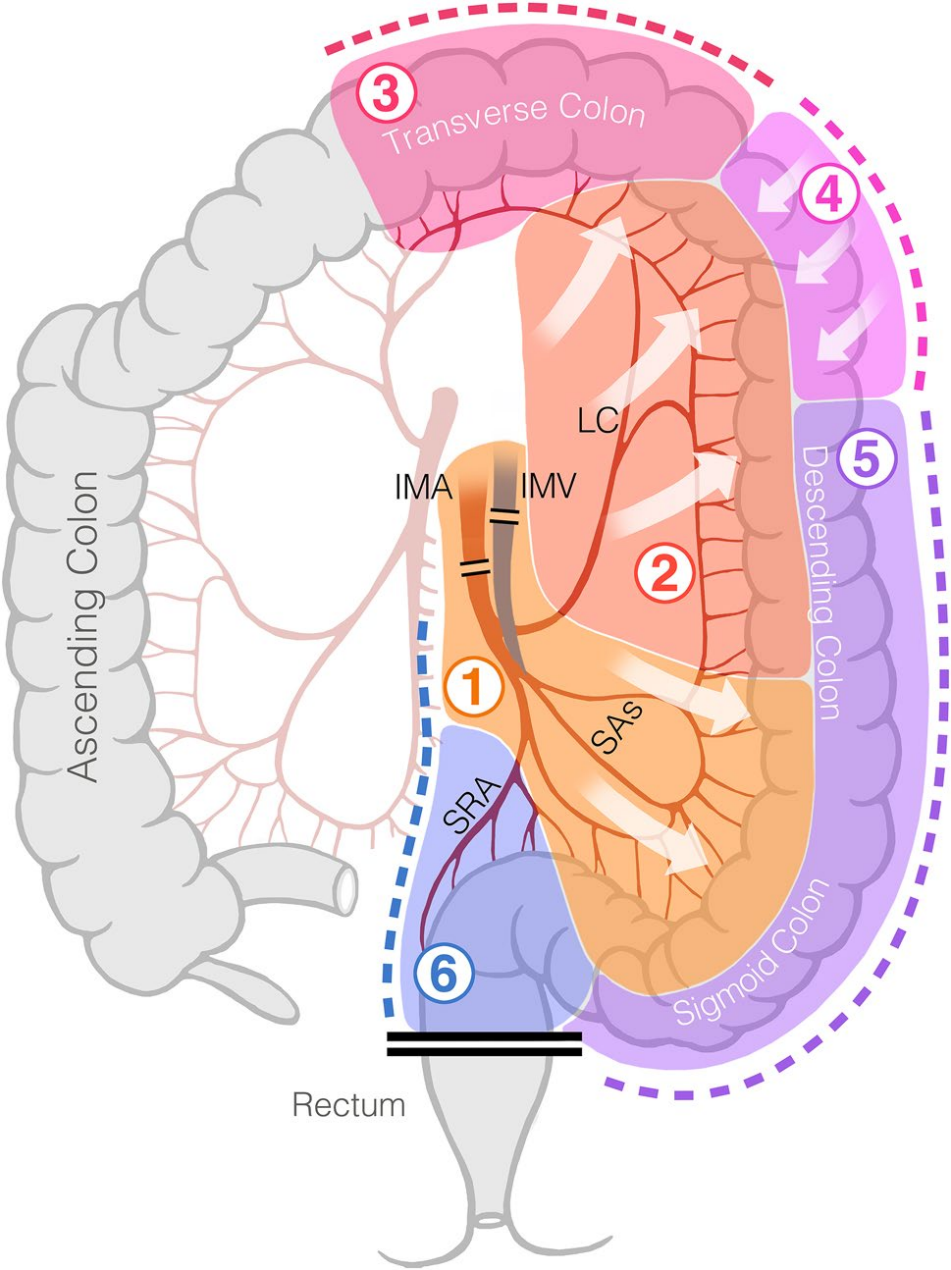
Description of the left colectomy study segments. Segment 1: dissection/division of IMA pedicle—division on or around IMA/IMV and within 2–3 cm of main vessels of mesentery (including window). Segment 2: retroperitoneal dissection—dissection in retroperitoneum above and below IMA/IMV, above the sigmoid up to splenic flexure posteriorly. Segment 3: omental dissection/resection—separation of omentum and mobilization of transverse colon up to the splenic flexure (not including splenic flexure). Segment 4: splenic flexure takedown—take down of splenic flexure with complete separation of it from retroperitoneum. Segment 5: lateral colonic dissection—lateral freeing up of sigmoid and left colon to everything below splenic flexure. Segment 6: mesenteric and pelvic dissection. *IMA* inferior mesenteric artery, *IMV* inferior mesenteric vein, *SAs* sigmoid arteries, *LC* left colic artery, *SRA* superior rectal artery

The primary outcomes were: (1) dissection time to specimen removal (DTSR), and (2) versatility score. The primary outcome DTSR was defined as time to specimen removal measured in minutes from the start of colon mobilization to specimen removal from the abdominal cavity. Versatility (composite of five variables) was measured by a score system from 1 to 5 (1 being worst and 5 the best) for each of the six specific segments (Fig. 1) and adjusted/weighted by coefficient of importance with distribution of the importance as follow: hemostasis 0.275, sealing 0.275, cutting 0.2, dissection 0.15, and tissue manipulation 0.1. A mean score of 3.5 and above was considered a high versatility and below 3.5 and lower as low versatility (4). The overall versatility score is presented as an average of the mean versatility scores from each of the six surgical segments each evaluated using the score displayed on Table 1. The Versatility score was developed before the trial and based on the surgeon’s experience about the relative importance of the five variables included in the versatility score.

**Table 1:** Versatility variables evaluation

Variable score	Variable definition	THUNDERBEAT	LigaSure
Hemostasis	Definition of score		
5	No bleeding at vessel or tissue site	5	5
4	Mild blood oozing at tissue site; no intervention needed	4	4
3	Moderate blood oozing at tissue site requiring intervention	3	3
2	Heavy bleeding requiring immediate further intervention	2	2
1	No hemostasis achieved with the instrument after two attempts	1	1
Sealing at surgery (visual score by surgeon)			
5	Complete seal using instrument 1 or 2 applications only on the named vessels	5	5
4	Complete seal but using instrument > 2 and < 4 times to seal the named vessels	4	4
3	Complete seal using instrument 4 and > 4 application less than 6 on the named vessels	3	3
2	Incomplete seal even after more than 6 applications on the named vessels	2	2
1	Incomplete seal, has to use another instrument to seal the named vessel	1	1
Cutting			
5	Complete tissue transection	5	5
4	Tissue transection but minor tissue strand remaining, not requiring reapplication of device	4	4
3	Tissue transection but tissue strand remaining requiring reapplication of device	3	3
2	Incomplete tissue transection multiple reapplication of device	2	2
1	No transection/cutting of tissue occurred, used another device to complete task	1	1
Dissection			
5	Excellent dissection capability. Separate tissues, no need from another instrument	5	5
4	Good. Able to dissect tissue but restricted jaw opening and/or ability to separate tissue	4	4
3	Average. Difficulties at ability to dissect tissue off structures	3	3
2	Fair. Limited jaw opening and/or ability to spread tissue	2	2
1	Unable to effectively dissect tissue off structures	1	1
Tissue manipulation			
5	Excellent manipulation capability. Grasps and manipulate tissue without any traumatic injuries	5	5
4	Good. Manipulates tissue but re-grasping occasionally without traumatic injuries or any injuries requiring repair	4	4
3	Average. Difficult to grasp and manipulate tissue. Traumatic injuries requiring repairs	3	3
2	Fair. Difficulties at grasp and manipulate tissue. Traumatic injuries requiring immediate repair	2	2
1	Unable to manipulate tissue and/or cause traumatic injuries requiring immediate repair	1	1

The ease of use of instruments was evaluated with a survey evaluating the surgeon’s opinion on the instrument immediately after surgery consisting of 8 questions on the scale from 1 to 10, where a score of 1 is worst and 10 is the best (Table 2). The secondary outcome, drier surgical field was an evaluation of the entire surgical field for overall oozing of blood or any other body fluids using a scoring system from 1 to 5 as described on Table 3.

**Table 2:** Surgeons’ instrument evaluation survey

#	Instrument	Score 1 worst to 10 best
	Device handling	
1	The ease of opening and closing the handle	1 2 3 4 5 6 7 8 9 10
2	Ease to maneuver	1 2 3 4 5 6 7 8 9 10
3	Weight balance	1 2 3 4 5 6 7 8 9 10
4	Fatigue from the use of handle, or any pain (1 = max pain, fatigue/10 = no pain fatigue)	1 2 3 4 5 6 7 8 9 10
5	The ease in pushing the handle seal and cut buttons	1 2 3 4 5 6 7 8 9 10
6	The ease of turning the rotor knob	1 2 3 4 5 6 7 8 9 10
7	What is the level of your confidence in sealing large vessels (more than 5 mm)	1 2 3 4 5 6 7 8 9 10
8	Overall satisfaction	1 2 3 4 5 6 7 8 9 10

**Table 3:** Drier surgical field: definitions and scores

Variable score	Variable definition	THUNDERBEAT	LigaSure
Drier surgical field	Definition of Score		
Variable score	Variable definition	THUNDERBEAT	LigaSure
Drier surgical score			
5	No oozing at vessel or tissue site in entire surgical field	5	5
4	Minimal/mild blood oozing at tissue site in 1 or 2 areas surgical field; no intervention needed	4	4
3	Moderate blood oozing at tissue site in few areas of the surgical field and requiring intervention	3	3
2	Heavy bleeding requiring immediate further intervention at any part of the surgical field	2	2
1	Heavy bleeding, hemostasis achieved with the instrument with more than two attempts	1	1

Other outcome data collected were failure of the energy instruments to control bleeding, complications related to use of the instrument, visible thermal spread, postoperative bleeding requiring intervention, thermal injuries manifestation after surgery, reoperation, readmission, length of hospital stay and mortality. Patients were assessed daily after surgery until discharge and at 30 days following surgery.

### Study instruments

#### THUNDERBEAT 5 mm to 35 cm (Olympus, Japan)

**Fig. 2:**
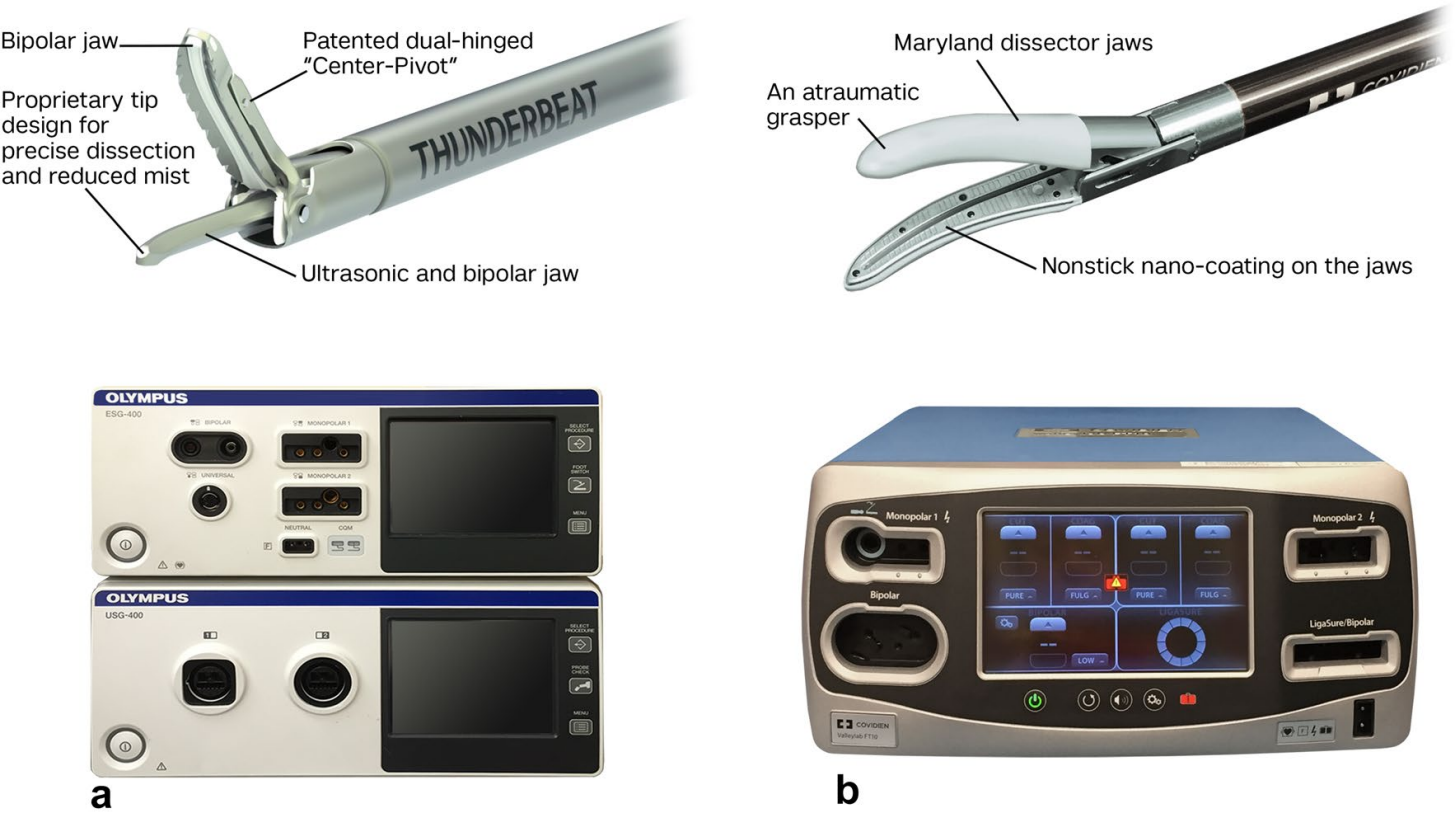
Energy devices **a** THUNDERBEAT (Olympus, Japan) and **b** Maryland LigaSure™ (Medtronic, USA)

The TB device has been cleared under 510 (K) by FDA and currently used for regular care. The surgeons were able to coagulate blood vessels up to 7 mm, cut and dissect during surgery. The device consists of: THUNDERBEAT device and generator (Fig. 2a). The device is provided sterile and intended for single use only. The generators settings were the same for all cases in this study.

#### Maryland LigaSure™ 5 mm to 37 cm (Medtronic, USA)

Maryland LigaSure device is an FDA approved device and currently is used throughout the USA in surgery for tissue dissection and vessel ligation. The device consists of the LigaSure device and Generator-Force Triad (Fig. 2b). The device is provided sterile and intended for single use only.

All participating surgeons were trained in using TB and Maryland LigaSure devices as they are used daily for regular surgical care, and surgeons used the devices on at least 10 patients prior to this study.

### Equipment

All surgeries were performed using the same laparoscopic equipment from Olympus and all cases were recorded with the Olympus video recording systems. The equipment included, High Definition LCD Monitor, Xenon Light Source, C02 Insufflator, laparoscopic camera 5 Endoeye Flex Deflectable scope, and Video System Center. Also, for all study cases the same THUNDERBEAT and Force Triad Generator were used with preset mode.

### Statistical considerations

Blocked randomization using the method of random permuted blocks was used in the trial. A series of randomized blocks of four was generated with a 1:1 allocation ratio to allow for an equal number of patients in the two groups, by independent statistician to minimize bias. Group status was defined by the two different instruments: Group 1 THUNDERBEAT and Group 2 LigaSure. The randomization was generated by a biostatistician, and subjects were assigned to the groups at the start of the surgery using randomization envelopes.

This study was designed as a pilot (exploratory) randomized study. Because at the time this study was planned there were no other studies in colorectal surgery evaluating THUNDERBEAT that could have been used for estimation of dissection time, the study was not powered to detect a specific difference in DTSR between the groups. Post hoc analysis was performed to compare the primary outcome DTSR between the groups.

The primary outcome DTSR between the groups was evaluated using Wilcoxon rank-sum test. Demographic, preoperative, and postoperative variables were compared between groups by the Wilcoxon rank-sum test for continuous variables and the *χ*^2^ test/Fisher’s exact test for categorical variables, as appropriate. The versatility score was calculated as defined. The surgeon’s opinion evaluation survey is presented as number and percentages, and then compared between the two instrument groups. All *p*-values are two-sided with statistical significance at the 0.05 *α* level. All analyses were performed in IBM SPSS Version 25 (Armonk, NY: IBM Corp).

## Results

For presenting the result in this manuscript, we used the CONSORT (Consolidated Standards of Reporting Trials) protocol guidance for more transparency and better quality of the report on RCT [13]. Seventy three patients were enrolled in the study from February 2016 to April 2019, and sixty of them were randomized into the two groups: *n* = 31 in Group 1: THUNDERBEAT and *n* = 29 in Group 2: LigaSure and included in the analyses. The other 13 patients were withdrawn from the study prior to the randomization for variety of reasons such as surgery cancellation, change of surgical plan or change in medical conditions excluding the subjects. All patients completed the 30 days follow up time. There were no statistical differences between the two groups in terms of demographics—age, sex, BMI, ASA, preoperative diagnosis, and preoperative comorbidities (Table 4). No significant difference was found between the groups in the primary outcomes, DTSR (*p* = 0.214) and the total time of the surgical procedure (*p* = 0.311). Detailed results of the intraoperative outcomes are listed in Table 5. Significant differences in versatility overall score and in surgical segment 3 versatility score were observed between the groups, with TB device having a higher score than LigaSure device (*p* = 0.045 and 0.041 respectively). In addition, TB demonstrated significantly higher scores in tissue dissection and tissue manipulation in surgical segment 3, and higher sealing score in segment 6 with *p* = 0.011, 0.026 and 0.023, correspondingly (Table 5). There were no significant differences between the groups regarding the coagulation tests PT, PTT, and INR (*p* = 0.463, 0.122, and 0.069, respectively).

**Table 4:** Results: Demographics and sample characteristics

Parameter	THUNDERBEAT *n* = 31	LigaSure *n* = 29	p*
Age in years median (range)	54 (31–92)	63 (29–88)	0.515
Female/Male	14/17	17/12	0.297
BMI mean/sd	26.3 ± 4.3	26.4 ± 4	0.965
ASA median (range)	2 (2–3)	2 (2–3)	0.923
Follow up time 30 days	31 (100%)	29 (100%)	
Preoperative diagnosis			
Neoplasm	11 (35.5%)	8 (27.6%)	0.511
Diverticulitis	20 (64.5%)	21 (72.4%)	0.511
Preoperative comorbidities			
HTN	8 (25.8%)	12 (41.4%)	0.201
CAD	4 (12.9%)	2 (6.9%)	0.438
COPD	2 (6.5%)	1 (3.4%)	0.594
Diabetes	1 (3.2%)	2 (6.9%)	0.514
Others	2 (6.5%)	1 (3.4)	0.514
Laparoscopic left colectomy	31 (100%)	29 (100%)	
Converted to open surgery	1 (3.2%)	2 (6.9%)	0.514

Demographic, preoperative, and postoperative variables were compared between groups by the Wilcoxon rank-sum test for continuous variables and the *χ*^2^ test/Fisher’s exact test for categorical variables, as appropriate

All *p*-values are two-sided with statistical significance evaluated at the 0.05 *α* level, *p < 0.05. *BMI* body mass index, *ASA* American Society of Anesthesiologists physical status classification, *HTN* hypertension, *CAD* coronary artery disease, *COPD* chronic obstructive pulmonay disease

**Table 5:** Results: Intraoperative outcomes

Parameter	THUNDERBEAT *n* = 31	LigaSure *n* = 29	p*
Intraoperative outcomes			
Dissection time to specimen removal min median/range	91 (41–172)	77 (38–175)	0.214
Length of surgical procedure min median/range	176 (113–270)	170 (98–265)	0.311
Lysis of adhesions min median/range	1 (0–70)	1 (0–42)	0.618
Versatility index scores mean/sd			
Overall versatility score mean/sd	4.8 ± 0.18	4.7 ± 0.22	0.045
Seg2 versatility score mean/sd	4.8 ± 0.33	4.7 ± 0.32	0.147
Seg3 versatility score mean/sd	4.9 ± 0.25	4.7 ± 0.39	0.041
Seg6 versatility score mean/sd	4.7 ± 0.30		0.070
Seg1 dissection score mean/sd	5 ± 0.02	4.8 ± 0.30	0.580
Seg2 hemostasis mean/sd	4.7 ± 0.60	4.4 ± 0.55	0.154
Seg2 dissection score mean/sd	5 ± 0.02	4.9 ± 0.41	0.147
Seg3 hemostasis mean/sd	4.6 ± 0.60	4.4 ± 0.61	0.168
Seg3 sealing mean/sd	4.9 ± 0.30	4.7 ± 0.51	0.085
Seg3 cut mean/sd	4.9 ± 0.18	4.8 ± 0.35	0.117
Seg3 dissection score mean/sd	4.9 ± 0.04	4.8 ± 0.38	0.011
Seg3 tissue manipulation score mean/sd	4.9 ± 0.03	4.8 ± 0.35	0.026
Seg4 dissection score mean/sd	5 ± 0.02	4.9 ± 0.30	0.067
Seg5 dissection score mean/sd	5 ± 0.02	4.9 ± 0.30	0.077
Seg6 hemostasis mean/sd	4.4 ± 0.65	4.2 ± 0.66	0.229
Seg6 sealing mean/sd	4.8 ± 0.40	4.4 ± 0.77	0.023
Seg6 dissection score mean/sd	5 ± 0.02	4.9 ± 0.26	0.101
Dryness of the surgical field average score mean/sd	4.5 ± 0.38	4.4 ± 0.33	0.572
Vessels sealing			
Number of applications until vessel coagulation achieved			
Left colic artery	2.2 ± 0.75	2 ± 1	0.930
Sigmoid arteries	2.4 ± 1.5	2.6 ± 1.3	0.469
Superior rectal arteries	2.7 ± 1.3	2.9 ± 1.2	0.316
IMA/IMV	3.1 ± 0.9	4.5 ± 3.7	0.866
Success of sealing	29 (93.5%)	27 (93.1%)	0.945
Bleeding at time of sealing	3 (9.7%)	4 (13.8%)	0.620
Bleeding mesentery dissection	4 (12.9%)	7 (24.1%)	0.261
EBL (ml)	87 ± 97	66.4 ± 64	0.419
Intraoperative blood transfusion	0	0	1
Intraoperative complications other except bleeding	0	1	0.760
Intraoperative complication related to the devices	0	0	1
Number of instruments exchanges in/out of abdomen	1.9 ± 1.2	1.38 ± 1.26	0.117

All *p*-values are two-sided with statistical significance evaluated at the 0.05 α level, *p < 0.05; *Seg* segment, *IMA* inferior mesenteric artery, *IMV* inferior mesenteric vein, *EBL* estimated blood loss in ml

Postoperatively, there were no significant differences between the groups in restoration of the gastrointestinal tract, postoperative complication within 30 days, and other hospital parameters of surgical recovery (Table 6). There was no mortality observed. Surgeon survey results are listed in Table 7. Surgeon’s overall satisfaction with the instrument was significantly higher for the THUNDERBEAT device (*p* = 0.015). LigaSure demonstrated a higher score for “less pain and less fatigue” from use the of instrument handle but it was not statistically significant (*p* = 0.08) (Table 7).

**Table 6:** Results: Postoperative outcomes

Parameter	THUNDERBEAT *n* = 31	LigaSure *n* = 29	p*	
GI recovery				
First flatus POD day median (range)	2 (1–4)	2 (1–4)	0.733	
First bowel movement POD day median (range)	2 (1–5)	2.5 (1–4)	0.835	
First solid food POD day median (range)	2 (1–8)	2 (1–4)	0.199	
Postoperative complications 30 days				Clavien–Dindo Grade
Delayed thermal injuries related to energy devices	0	0	1	–
Postoperative rectal bleeding	3 (9.7%)	4 (13.8%)	0.620	Grade IIIa-1
				Grade IIIb-6
Anastomotic leak	0	2 (6.9%)	0.137	Grade IIIa-1
				Grade IIIb-1
Postoperative ileus	1 (3.2%)	0	0.329	Grade II-1
Wound hematoma incision	1 (3.2%)	3 (10.3)	0.269	Grade I-4
UTI	1 (3.2%)	0	0.329	
Hospital parameters				
LOHS in day mean/sd	4.5 ± 3.2	5.3 ± 3.9	0.272	
LOSS in days mean/sd	4.5 ± 3.1	5.3 ± 3.9	0.265	
Reoperation	2 (6.5%)	4 (13.8%)	0.344	
Readmission	1 (3.2%)	0	0.329	
Mortality	0	0	1	

All *p*-values are two-sided with statistical significance evaluated at the 0.05 α level, *p < 0.05

*LOHS* length of hospital stay from admission to hospital discharge, *LOSS* length of postsurgical stay from day of surgery to hospital discharge, *UTI* urinary tract infection

**Table 7:** Results: Surgeons survey

Instrument	THUNDERBEAT *n* = 31	LigaSure *n* = 29	*p*
Device handling			
Ease of opening and closing the handle	8.5 ± 1	7.7 ± 1.2	0.012
Ease to maneuver	8.5 ± 0.9	7.7 ± 1.1	< 0.0001
Weight balance	8.6 ± 0.7	8.3 ± 0.9	0.104
Fatigue from the use of handle, or any pain (1 = max pain, fatigue/10 = no pain fatigue)	8.5 ± 1.6	9.2 ± 1.1	0.08
The ease in pushing the handle seal and cut buttons	8.2 ± 1.3	7.2 ± 1.5	0.007
Ease of turning the rotor knob	7.6 ± 1.6	7.0 ± 1.4	0.089
What is the level of your confidence in sealing large vessels (more than 5 mm)	8.8 ± 1.5	8.4 ± 1.6	0.31
Overall satisfaction	8.5 ± 1.1	7.8 ± 1	0.015

## Discussion

The goal of this randomized trial was to evaluate the clinical performance between TB and Maryland LigaSure Energy Devices with primary outcome DTSR. Surgical time has become an important intraoperative factor along with safe and effective hemostasis and tissue dissection, as it is directly related to the cost-effectiveness of the surgical procedure. Development of new advanced energy based surgical instruments for laparoscopic surgery have shortened the surgical procedure time and have replaced the conventional hemostasis tools such as sutures, clips and staples with fast, safe and effective hemostasis. Our study did not find significant difference between the TB and LigaSure devices, consistent with the finding of other recent publications [2, 12]. A retrospective study from Italy compared outcomes and cost between TB and three other energy-based devices in patients undergoing laparoscopic colorectal resection, did not find any significant difference in the operative time and other outcomes between the groups [2]. Similar findings were reported by Shuradja in 2018 from a prospective study with retrospective cohort for comparison of TB to LigaSure and Harmonic Ace. The study reported no significant difference between the devices in operative time and suggested that they are equally safe and effective [12]. In our study, the 12-min difference in DTSR between the groups (higher in TB group) was neither clinically nor statistically significant. A post hoc power calculation shows that a statistically significant 12-min difference between the two groups could only be detected with a power of 24%. The current study, with 30 patients per group, if adequately powered at 80%, would be able to detect a difference of 27 min or greater between the two groups. Nonetheless, since 12 min may affect the cost in the operating room, the effect of the surgeon’s experience with the energy device on the operative time was evaluated. We found significantly less time to specimen removal between one experienced surgeon compared to the rest of the surgeons, which may explain the 12 min difference between the groups. Since there is no significant difference between the devices in the time to specimen removal and the overall total time of the surgery, number of applications until vessel coagulation achieved, and number of instruments exchanges in/out of the abdomen, we did not conduct a cost analysis. The actual cost between the devices is also similar (TB $475 vs. LigaSure $495). However, the device prices may differ between different institutions. Allaix et al. also did not find any cost difference between TB and other energy devices [11].

The versatility of the new surgical devices allowing multiple functions of the device is important for shortening the surgical time and minimizing the instruments exchange while providing safe and effective tissue dissection and vessel sealing. In order to have more objective evaluation of the clinical usefulness of the energy devices in the study, we used a “versatility score” (see methods) [4, 5]. In this study, the TB demonstrated significantly higher overall versatility score vs. LigaSure (4.8 vs. 4.7, *p* = 0.045), clinically inconsequential, as both instruments had high versatility scores. Two previous studies have reported significantly higher versatility score for TB device compared to other surgical devices, but this versatility difference was not reported to affect the clinical performance of the devices [4, 5].

The TB tip (Fig. 2a, b) allows making a swift entry into a new surgical plane, which is a technical differentiation between it and the LigaSure. Both instruments demonstrated safe and effective clinical performance in vessel sealing of left colic artery, sigmoid arteries, superior rectal arteries and large vessels such as IMA and IMV. There was no significant difference in the number of applications until vessel coagulation was achieved for sealing of all vessels including IMA and IMV. In two cases, with calcified vessels (one in TB group and one in LigaSure group), the numbers of applications were higher, and clips had to be used to achieve vessel sealing and hemostasis. This is an important concept in all compression energy devices.

One of the main concerns when working with ultrasonic energy is thermal spread. With TB and other energy devices like Harmonic Ace (Johnson & Johnson), there is a high temperature at the vibrating jaws, which can reach up 200 °C compared to 100 °C in bipolar instruments, theoretically increasing the risk of thermal injuries [4, 14]. Seehofer et al. using a Thermocamera reported that after repeated activation TB reaches 172 ± 7 °C, and 60 seconds are needed to decline to a safer 60 C [15, 16]. Despite these data, no intraoperative or postoperative delayed thermal injuries occurred in our study.

This study also evaluated the “dryness” of the surgical field, assessing for generalized oozing during mesenteric and vessel dissection, and lysis of adhesions [17]. Both groups had similar scores (Table 5). Patient’s coagulation factors PT, PTT and INR were evaluated prior to surgery, and patients on anticoagulants prior to surgery or coagulopathy disorders were excluded from the study in order not to affect the instruments hemostasis and sealing evaluation. During surgery all patients had warm air blanket and were well monitored for hypothermia.

Postoperatively, the groups did not differ significantly in complication rates. Three patients in Group 1 and four in Group 2 experienced rectal bleeding not related to the energy devices but rather to the intestinal anastomosis. No patients experienced abdominal bleeding postoperatively requiring reoperation. One patient required transfusion postoperatively following rectal bleeding from their anastomosis. Two patients were treated for anastomotic leak and recovered successfully, with one requiring reoperation and temporary stoma. Neither event appeared to have any relation to use of an energy device.

**Fig. 3:**
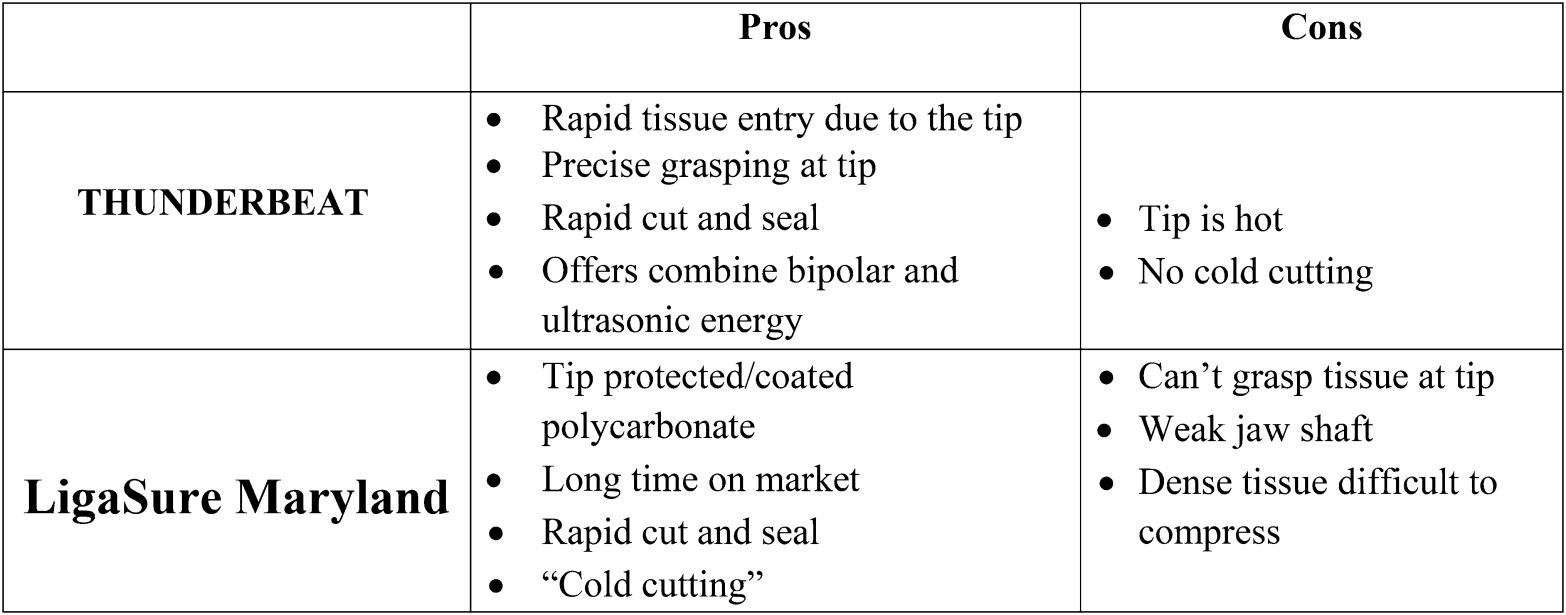
Useful tips for technical aspects of using THUNDERBEAT and LigaSure

Surgeon’s overall satisfaction with the instruments showed a significant preference for the TB regarding ease of opening and closing the handle, ease to maneuver the instrument, and the ease in pushing the handle seal and cut buttons (Table 7). Less fatigue and pain were reported with the LigaSure, but it was not statistically significant. While the survey was designed to evaluate the surgeon’s satisfaction with the energy devices, the results are surely influenced by the surgeon’s individual preference and experience, as only five surgeons participated in the study and half of the patients were operated on by one experienced surgeon. Therefore, the significant difference between the groups should not be interpreted as clinically important or be generalized to other surgeons’ experience with LigaSure or TB energy devices. However, this information may be useful for improvement of the device’s features. A few pros and cons about the technical aspect of the devices are presented in Fig. 3.

Limitations of the study include the following:

1. It was conducted in a single academic institution with highly experienced colorectal surgeons in laparoscopy and surgical energy devices.
2. The study used the Maryland LigaSure Energy Device for comparison, which also was a new device at that time this study was designed. It is possible that other style LigaSure energy devices (e.g., blunt tipped) may have performed better than the one used in this study.
3. At the time we embarked on this study, there were no other studies in colorectal surgery evaluating THUNDERBEAT to use for estimation of dissection time. Post hoc analysis was performed to compare the DTSR between the groups, thus the results from this study may not be generalizable.

In summary, the TB and LigaSure devices compared in this randomized trial did not show significant difference between the study groups in the primary outcome, time to specimen removal. No patient in either group had any complication related to the energy devices, most importantly bleeding or thermal injury. A versatility score comparing the instruments is significantly different favoring TB, but not clinically significant as both groups have very high versatility scores. The study did not find any significant difference in intraoperative or postoperative complications or any complications related to the energy devices.

*In conclusion*, in a randomized control trial comparing THUNDERBEAT and LigaSure compressive energy devices, both were effective and safe in dividing soft tissue and sealing mesenteric blood vessels during laparoscopic left colon surgery, with THUNDERBEAT demonstrating some advantages over LigaSure during omental dissection and tissue manipulation.
